# 20(S)-Ginsenoside Rh2 Induce the Apoptosis and Autophagy in U937 and K562 Cells

**DOI:** 10.3390/nu10030328

**Published:** 2018-03-07

**Authors:** Jianjian Zhuang, Juxin Yin, Chaojian Xu, Ying Mu, Shaowu Lv

**Affiliations:** 1 Key Laboratory for Molecular Enzymology and Engineering of the Ministry of Education, College of Life Science, Jilin University, Changchun 130000, China; zhuangjianjian1234@163.com (J.Z.); cavalierxu@yeah.net (C.X.); 2 State Key Laboratory of Industrial Control Technology, Research Center for Analytical Instrumentation, Institute of Cyber-Systems and Control, Zhejiang University, Hangzhou 310000, China; yinjuxin@163.com (J.Y.); muying@zju.edu.cn (Y.M.)

**Keywords:** 20(S)-GRh2, AML, CML, apoptosis, autophagy, cooperative manner

## Abstract

Acute myeloid leukemia (AML) and Chronic myelogenous leukemia (CML) are common leukemia in adults. 20(S)-GRh2 is an important bioactive substance that is present in Panax ginseng. However, there are no investigations that deal with the comparison of apoptosis, the occurrence of autophagy, and the relationship between apoptosis and autophagy after being treated with 20(S)-GRh2 in AML and CML. In this study, we explored the effect of 20(S)-GRh2 on the AML and CML (U937 and K562). Fluorescence microscopy, CCK-8, Quantitative realtime PCR, Western blot, transmission electron microscopy (TEM), and flow cytometric analysis were used to detect the occurrence of cell proliferation inhibition, apoptosis, and autophagy. By using the above methods, it was determined that apoptosis induced by 20(S)-GRh2 was more obvious in K562 than U937 cells and 20(S)-GRh2 could generate autophagy in K562 and U937 cells. When pretreated by a specific inhibitor of autophagy, (3-methyladenine), the 20(S)-GRh2-induced apoptosis was enhanced, which indicated that 20(S)-GRh2-induced autophagy may protect U937 and K562 cells from undergoing apoptotic cell death. On the other hand, pretreated by an apoptosis suppressor (Z-VAD-FMK), it greatly induced the autophagy and partially prevented 20(S)-GRh2 induced apoptosis. This phenomenon indicated that 20(S)-GRh2-induced autophagy may serve as a survival mechanism and apoptosis and autophagy could act as partners to induce cell death in a cooperative manner. These findings may provide a rationale for future clinical application by using 20(S)-GRh2 combined autophagy inhibitors for AML and CML.

## 1. Introduction

Acute myeloid leukemia (AML) and Chronic myelogenous leukemia (CML) are common leukemia malignant blood diseases [1], and an uncontrolled proliferation, different clinical manifestation, and the accumulation of deviant hematopoietic progenitor cells are the characteristics of the lethal hematological malignancy that originated from myeloid progenitor cells [2]. AML, if not treated timely, will cause patients to die within six months. As once CML develops beyond its control, patients usually live for only four years [3]. 

Many drugs are explored for the treatment of leukemia. However, standard therapies including the commonly employed combination chemotherapy have some side effects, both in short and long term [4,5]. Daunorubicin in combination with cytarabine chemotherapy is a commonly used regimen, however, there is an increased risk of toxic effects of myelosuppression and infection with prolonged treatment. At the same time, elderly patients cannot tolerate high-dosechemotherapy [6,7]. In order to avoid the serious side effects resulting from chemotherapy and to improve the survival rates, newer anticancer drugs are continuously being explored.

Chinese herbal medicines have the characteristics of low toxicity, and thus have a wide range of applications in the treatment of cancer [8,9,10]. Panax ginseng is an important herbal product, which has an enormous medicinal effect and numerous studies have shown that ginseng has a positive effect on cancer prevention and treatment and can improve the well-being of patients during cancer treatment [11,12]. Related research has also confirmed that a regular consumption of ginseng preparations can prevent the cancer of mouth, stomach, lung, liver, pancreas, ovary, andcolon [9,13]. Ginsenosides are the group of active substances in ginseng that play a key role in the treatment of cancer, and, in particular, Ginsenoside Rh2 (GRh2) from this group has a pronounced activity [14,15,16]. GRh2 belongs to the protopanaxadiol family, which has two stereoisomeric forms, 20(S) and 20(R). When compared to 20(R)-GRh2, 20(S)-GRh2 form shows more evident anticancer activity through suppressing the cell proliferation [17]. 20(S)-GRh2 could induce apoptosis triggered through the mitochondrial pathway in human acute leukemia cells [18,19,20]. Liu et al. reported that 20(S)-GRh2 could inhibit the proliferation of myeloid leukemia cell lines, such as K562 and KG1a, by reducing the expression and activity of HDACs, increasing the acetylation of histones, and regulating the key proteins in the downstream signaling pathways [21]. Wang et al. reported that 20(S)-Rh2-induced apoptosis and participated in the Nur77-mediated signaling pathway, which could result in the exposure of BH3 domain of Bcl-2 activation of Bax in HL60 cells [22]. Wang et al. revealed that 20(S)-GRh2, in HepG2 and HEK-293T cells, could promote apoptosis by combining with Annexin A2 to down-regulate NF-кB activation and anti-apoptosis gene expression [23]. In recent years, research investigation focusing on studying the relationship between apoptosis and autophagy are attracting intensive attention in cancer therapy [24,25,26]. Autophagy and apoptosis are catabolic pathways that are essential for the homeostasis of organisms, where both involved in the ultimate fate of cancer cells [27]. Autophagy and apoptosis have an intricate relationship that varies with cells and stress [28], and which determines whether a cell will live or die in response to anticancer therapies [29]. Fang et al. studied the relationship between apoptosis and autophagy in 20(S)-GRh2-treated ALL cells and found that autophagy plays a protective role in 20(S)-GRh2-induced apoptosis [18]. Cho et al. reported that ginsenoside F2 induces apoptotic cell death accompanied with protective autophagy in breast CSCs [30]. 

The human CML cell line K562 has been used extensively as a model for the study of leukemia and the BCR-ABL fusion gene that it carries leads to uncontrolled cell proliferation [31,32,33,34]. U937, which belongs to AML-M5, has been extensively used as an in vitro model and is linked with complex mixed-series leukemia gene and the most frequently recurring cytogeneticabnormalities [35]. So far, in the current investigations of related to of 20(S)-GRh2 in U937 and K562 cells, there are no studies that have been reported on the occurrence of autophagy, the comparison of apoptosis, and the relationship between apoptosis and autophagy in U937 and K562 cells induced by 20(S)-GRh2. In this study, for the first time, we investigated the comparison of apoptosis in K562 and U937 cells. Moreover, we found out that the occurrence of autophagy in the 20(S)-GRh2-treated U937 and K562 cells. Finally, the relationship between autophagy and apoptosis has been reported. The outcome that was obtained through our investigations provides an important basis to clarify the mechanisms underlying the activity of 20(S)-GRh2 in inhibiting the growth of U937 and K562 cells. These findings provide a rationale for future clinical application by using 20(S)-GRh2 combined autophagy inhibitors for AML and CML.

## 2. Experimental

### 2.1. Materials

20(S)-GRh2 (99.48% purity) and Z-VAD-FMK and 3-MA were purchased from Sigma (Sigma Chemical Co., St. Louis, MO, USA) Roswell Park Memorial Institute (RPMI) 1640 medium was obtained from Gibco (Gibco, Rockville, MD, USA). 10% fetal bovine serum (FBS) was from HyClone (Logan, UT, USA). Cell Counting Kit-8 (CCK-8), Hoechst stain Kit, Annexin-FITC Apoptosis Kit, caspase-3 Activity Assay Kit, and Braford assay Kit were obtained from Dojindo Laboratory (Dojindo Laboratory, Kumamoto, Japan). MDC stain kit was received from Sigma (Sigma Chemical Co., St. Louis, MO, USA). Cellular Reactive Oxygen Species Detection Assay Kit was from Beyotime Biotechnology Inc. (Beyotime Biotechnology Inc., Shanghai, China). Mitochondrial Membrane Potential Assay Kit and caspase-9 Fluorescence Metric activity assay (KGA402F-KGA404F) were from R&D Systems (R&D Systems, Minneapolis, MN, USA). RNA Isolation kit, Reverse transcription kits, and SYBR Premix Ex Taq™ kit were from Takara Biotechnology (Takara Biotechnology Co., Ltd., Tokyo, Japan). Polyclonal rabbit anti-human cleaved caspase-3 (9661T), PARP (#9542), p62 (#5114), LC-3B (12741T) antibody, and secondary HRP-conjugated anti-rabbit antibody (#7074) were received from CST (Boston, MA, USA).

### 2.2. Cell Culture

U937 and K562 cell lines were cultured at a density of 1 × 10^5^ cells/mL at 37 °C, in the presence of 5% CO_2_ and in a humidified atmosphere. Both cells were maintained in Roswell Park Memorial Institute (RPMI) 1640 medium supplemented with 10% fetal bovine serum (FBS) and 100 units/mL of Penicillin and 100 μg/mL of Streptomycin. 

### 2.3. Hoechst 33258 Staining Assay 

Cells treated with 20(S)-GRh2 for 24 h, and after the treatment they were collected and rinsed in PBS. Then, the cells were stained with Hoechst 33342 for 30 min in the dark. Observations were then made through fluorescence microscopy (Olympus, Tokyo, Japan). Normal and apoptosis cells were counted in five randomly chosen fields to calculate the percentage of apoptosis cells at each time interval.

### 2.4. Growth Inhibition Assay 

The viability of U937 and K562 cell lines were assessed by using Cell Counting Kit-8 (CCK-8, Bestbio, Shanghai, China). U937 and K562 cell lines with a density of 1 × 10^4^ cells were seeded in 96-well plates at 37 °C in a humidified atmosphere of 5% CO_2_. U937 and K562 cell lines were treated with 20(S)-GRh2 at 20 μmol/L, 40 μmol/L, 60 μmol/L, 80 μmol/L and 100 μmol/L for 24 h. Control cells were cultured in 0.5% DMSO medium only. At the end of experiments, 10 μL of CCK-8 was added to each well and incubated for an additional 4 h at 37 °C in 5% CO_2_. The absorbance at 450 nm was measured by using ELX 808 microplate reader (Bio-Tek Instruments, Bad Friedrichshall, Germany).

### 2.5. Apoptosis Analysis

The U937 and K562 cells for apoptosis were examined using Annexin V-FITC Apoptosis Detection Kit (Besbio, Shanghai, China). U937 and K562 cell lines were seeded at a density of 5 × 10^5^ cells/well in 96-well plates. The U937 and K562 cell lines were treated by 60 μmol and 80 μmol/L 20(S)-GRh2 for 24 h. Control cells received an equal amount of 0.5% DMSO at equal time. After treatment, the cells were collected and washed with cold PBS. The cell pellets were then incubated in 500 µL Annexin V binding buffer containing 2 µL of FITC-Annexin V for 15 min, and were then resuspended with propidium iodide in dark at 4 °C for 5 min. The fluorescence intensity was measured using flow cytometry for Annexin V-FITC (with the excitation wavelength at 488 nm and emission wavelength at 525 nm) and PI (with the excitation wavelength at 488 nm and emission wavelength at 620 nm). The apoptosis ratios were analyzed using the Cell Quest software (Becton Dickinson, San Jose, CA, USA).

### 2.6. ROS Determination

The Cellular ROS Detection Assay Kit (Beyotime Biotechnology Inc., Shanghai, China) was used to determine the influence of 20(S)-GRh2 on the production of ROS levels. H_2_DCFDA was used as the cell permeable fluorescent probe, which is hydrolyzed by cellular esterase to non-fluorescent form and was oxidized to fluorescent DCF in the presence of ROS. Briefly, the U937 and K562 cell lines were seeded in 96-well plates at a density of 1 × 10^6^ cells/well. The cells were treated by 60 μmol/L and 80 μmol/L of 20(S)-GRh2 for 24 h. Control cells received an equal amount of 0.5% DMSO at an equal time. After 24 h of treatment, the cells were incubated with 10 mM of H_2_DCFDA (1:1000 dilution) for 30 min at 37 °C. The fluorescence of ROS was measured using BD flow cytometry with the excitation wavelength at 488 nm and emission wavelength at 525 nm.

### 2.7. Mitochondrial Membrane Potential (MMP) Assessment

Mitochondrial membrane potential (MMP) is an important parameter of mitochondrial function and the loss of ∆ψm may be an early event in the process of apoptosis of cells. The membrane potential was measured by using JC-1 Mitochondrial Potential Detection Kit (Jiangsu KeyGen BioTech Co., Ltd., Jiangsu, China). The U937 and K562 cells were seeded at a density of 1 × 10^6^ cells/well in 96-well plates, where the control cells received an equal amount of 0.5% DMSO. After 24 h of treatment, the cells were washed with cold PBS and were stained with JC-1 for 30 min at 37 °C in the dark. The suspension of cells was filtered through 400-mesh nylon, and was measured by FACScan flow cytometry with the excitation wavelength at 488 nm and emission wavelength at 525 nm.

### 2.8. Quantification of Apoptosis and Autophagy Related Gene by qRT-PCR

The expression of mRNA was measured by a quantitative reverse transcriptase PCR (qRT-PCR). U937 and K562 cell lines were seeded into 35 mm culture plates at a density of 1 × 10^6^ cells/well. The cells were treated with 60 μmol/L and 80 μmol/L of 20(S)-GRh2. Control cells received an equal amount of 0.5% DMSO. The cells were collected and washed by cold PBS. RNA was extracted from cells by using an RNA Isolation kit (Takara, Tokyo, Japan). The purity of RNA was measured by Nanodrop (NanoDrop Technologies, Wilmington, DE, USA) and A260/A280 ≥ 1.8. Then, RNA was reverse transcribed to cDNA by using Reverse transcription kits (Takara, Tokyo, Japan). The designed PCR primers were as follows: bax forward, 5′GGAGGAAGTCCAATGTCCAG3′ and reverse, 5′GGGTTGTCGCCCTTTTCTAC3′; bcl-2 forward, 5′GAGAAATCAAACAGAGGCCG3′ and reverse, 5′CTGAGTACCTGAACCGGCA3′; bcl-X_L_ forward, 5′CTGCTGCATTGTTCCCATAG3′ and reverse, 5′TTCAGTGACCTGACATCCCA3′; caspase-3 forward, 5′CTGCCTCTTCCCCCATTCT3′ and reverse, 5′TCGCTTCCATGTATGATCTTTG3′; caspase-9 forward, 5′AGGTTCTCAGACCGGAAACA3′ and reverse, 5′CTGCATTTCCCCTCAAACTC3′; Beclin-1 forward, 5′CTCCTGGGTCTCTCCTGGTT3′ and reverse, 5′TGGACACGAGTTTCAAGATCC3′; ATG5 forward, 5′GCCATCAATCGGAAACTCAT3′ and reverse, 5′ACTGTCCATCTGCAGCCAC3′; ATG7 forward, 5′ATTGCTGCATCAAGAAACCC3′ and reverse, 5′GAGAAGTCAGCCCCACAGC3′; LC3B forward, 5′GAGAAGACCTTCAAGCAGCG3′ and reverse, 5′TATCACCGGGATTTTGGTTG3′; β-actin forward, 5′TGACGTGGACATCCGCAAAG3′ and reverse, 5′CTGGAAGGTGGACAGCGAGG3′; The amplification system was performed by using a SYBR Premix Ex Taq™ kit (Takara, Tokyo, Japan). The qRT-PCR was performed by using the ABI Prism 7500 Sequence Detection System (Applied Biosystems, Carlsbad, CA, USA) and by employing the following experimental conditions: an initial denaturation at 95 °C for 3 s, followed by 40 cycles of amplification, and 60 °C for 34 s for data collection. The data were calculated relative to a calibrator using the formula, 2^−△△Ct^.

### 2.9. Caspase-9, Caspase-3 Activity Assay

The activities of caspase-9 and caspase-3 proteins were measured by caspase-9, caspase-3 Activity Assay Kit (BestBio, Shanghai, China). U937 and K562 cell lines were seeded into 100 mm culture plates at a density of 1 × 10^6^ cells/well. The cells were treated by 60 μmol/L and 80 μmol/L of 20(S)-GRh2. Control cells received an equal amount of 0.5% DMSO. The cells were collected and washed with cold PBS. The cell pellets were lysed and the equal amounts of cell lysate were co-cultured with specific fluorogenic substrate. The activation of caspase-3 (with the excitation wavelength at 400 nm and emission wavelength at 505 nm) and caspase-9 (with the excitation wavelength at 485 nm and emission wavelength at 535 nm) were measured by fluorescence enzyme microplate reader. The ratio of the experimental and control groups was presented.

### 2.10. Monodansylcadaverine (MDC) Staining Assay for Autophagy Detection

MDC, which is a lysosomotropic fluorescent compound, is usually used to detect the formation of autophagic bodies. The U937 and K562 cells were treated by 60 μmol/L of 20(S)-GRh2 for 24 h. Control cells received an equal amount of 0.5% DMSO. After incubation, the cells were stained with MDC for 30 min. Autophagosomes were observed with a fluorescence microscope (Olympus, Tokyo, Japan) at the excitation wavelength of 355 nm and using an emission wavelength of 512 nm.

### 2.11. Transmission Electron Microscopy (TEM)

U938 and K562 cells were seeded into 96-well plates and were subjected to respective treatments. After 24 h of treatment, the cells were collected and washed with PBS (pH 7.4). Then, the cells were fixed with 2.5% phosphate-buffered glutaraldehyde for 1 h at 4 °C prior to embedding. After three washes with PBS, the cells were post-fixed with 1% osmium tetroxide (OsO_4_) for 30 min, dehydrated with an increasing gradient of ethanol and acetone, and embedded into epoxy resin. Ultrathin sections (60–80 nm) were obtained with a diamond knife on a Leica Ultracut UCT (Leica Microsystems GmbH, Wetzlar, Germany), adhered to uncoated 200-mesh copper grids, stained with 2% uranyl acetate and lead citrate for 15 min each, and then observed under TEM (JEM-1400/JEM-1400 PLUS, Tokyo, Japan) at 80 kV.

### 2.12. Western Blotting Analysis

Western blot was used to detect apoptosis and autophagy related proteins. After treatment, the cells were collected and washed with cold PBS, lysed in RIPA buffer containing 1 mM PMSF, the lysates were then centrifuged at 12,000× *g* for 15 min at 4 °C. The quantification of total protein was made by using a BCA Protein Assay Kit (BestBio, Shanghai, China) and was separated by 10–15% SDS-PAGE, which was then transferred to polyvinylidene difluoride membranes (Millipore, Temecula, CA, USA). The membranes were blocked with 5% non-fat dry milk in PBS-Tween 20 for 2 h, and were incubated with a polyclonal rabbit anti-human cleaved-caspase3, PARP, p62, LC3B antibody (1:400) at 4 °C overnight. The membranes were incubated with a secondary HRP-conjugated anti-rabbit antibody (1:1000) for 2 h. The immunoreactivity bands were visualized by chemiluminescence.

### 2.13. Statistical Analysis

The results have been represented as the means ± standard deviation (SD). Statistical significance was carried out by the analysis of variance (ANOVA) test, followed by Newman-Keuls multiple comparison test (GraphPad Prism 3.0, GraphPad Software, San Diego, CA, USA). *p* < 0.05 was considered to be statistically significant. All of the experiments were performed in triplicate.

## 3. Results 

### 3.1. 20(S)-GRh2 Inhibits Proliferation of Myeloid Leukemia Cell Lines through Apoptotic Cell Death

**Figure 1:**
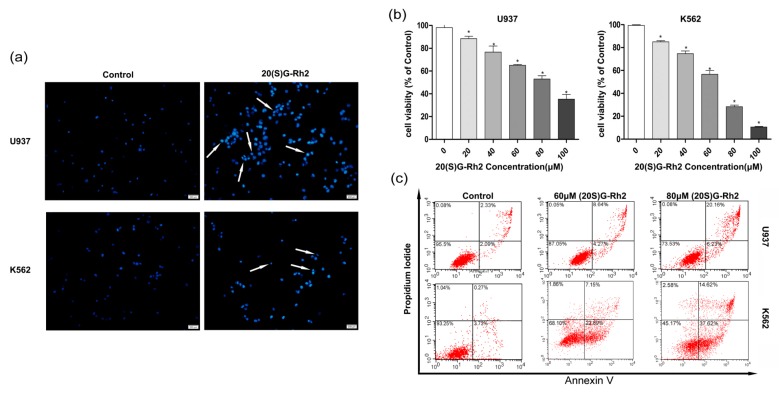
Apoptotic effects of 20(S)-GRh2. (**a**) Hoechst 33342 staining of U937 and K562 leukemic cells treated with 20(S)-GRh2 for 24 h. The apoptosis is characterized by chromatin condensation and nuclear fragmentation. (Scale bar: 50 μm); (**b**) CCK-8 assay was used to verify the proliferation inhibition of 20(S)-GRh2 in U937 and K562 cells; (**c**) Flow cytometry was used to detect the apoptotic ratio under 20(S)-GRh2 in U937 and K562 cells. The total apoptosis ratios are including apparent early apoptosis (lower right (LR) quadrant) and late apoptosis (upper right (UR)). The U937 and K562 cells were treated by 60 μmol and 80 μmol/20(S)-GRh2. Control cells received an equal amount of 0.5% DMSO. Values shown in (**b**) are mean ± SD (**a**–**c**) are the representative experiment with *n* = 3 technical replicates from two independent experiments with similar results. * *p* < 0.05 and vs. control group.

To explore the cell proliferation effects of 20(S)-GRh2 on myeloid leukemia, the assessment of its dose dependent effects was carried out using myeloid leukemia (AML cell types U937, CML cell types K562) cell lines. The Hoechst 33342 staining was used to study the morphological changes of apoptotic cells. Figure 1a shows a higher nuclear fragment and chromatin condensation in U937 and K562 cells when treated with 20(S)-GRh2. The effect of 20(S)-GRh2 on cell viability in leukemia cell lines was investigated by cell counting kit-8 (CCK-8) assay, whereby the obtained results showed that 20(S)-GRh2 significantly reduced the viability of U937 and K562 cells in a dose-dependent manner (Figure 1b). The IC50 of 20(S)-GRh2 was about 80 μM for U937 cells and 60 μM for K562 cells. To determine the proliferation inhibition of 20(S)-GRh2, the apoptosis in U937 and K562 cells was further examined. The Annexin-V and PI assays were used to distinguish between early apoptosis (lower right quadrants) and late apoptotic or necrotic cells (upper right quadrants), and the obtained results have been represented by the apoptosis ratio. The apoptotic ratios are estimated by the sum of number proportions of the early (the lower right quadrant) and late apoptotic cells (the upper right quadrant) to total cells tested [36], and have been shown in Figure 1c. 60 μM (80 μM) of 20(S)-GRh2 resulted into an apoptosis ratio of 12.91% (26.39%) in U937 cells and 30.04% (52.24%) in K562 cells, which indicate an increasing apoptosis in a dose-dependent manner. 

### 3.2. 20(S)-GRh2 Treatment Induces Cells Apoptosis through Mitochondrial Apoptosis Pathway

**Figure 2:**
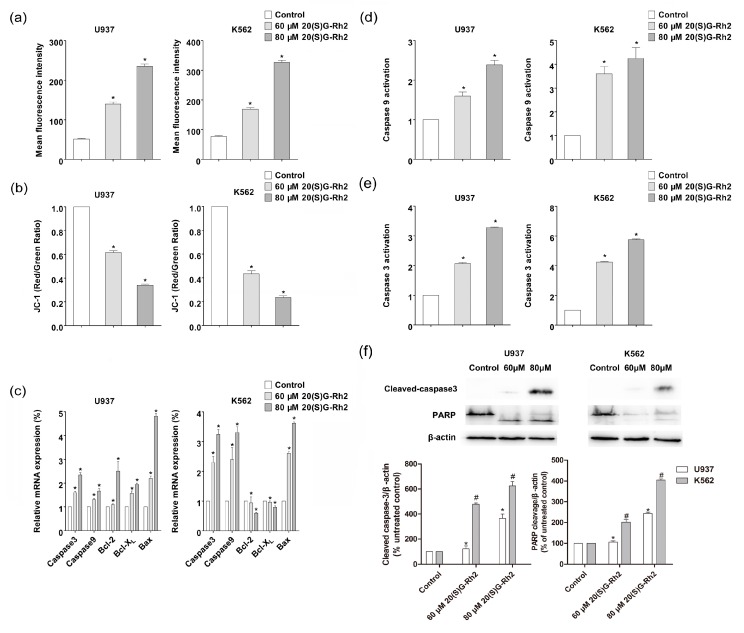
20(S)-GRh2 induced mitochondrial apoptosis in U937 and K562 cells. (**a**) The production of intracellular ROS level was detected by H_2_DCFDA fluorescent probe. The cells incubated with 60 μmol/L and 80 μmol/L of 20(S)-GRh2. After 24 h of treatment, the cells were incubated with H_2_DCFDA for 30 min. The contents of ROS were calculated by measuring the fluorescence intensity of DCF; (**b**) The loss of MMP via JC-1 staining was analyzed by FACScan flow cytometry. The treated cells were incubated with JC-1 for 30 min. The decreasing relative proportion of red and green fluorescence represents the loss of mitochondrial membrane potential (MMP); (**c**) The expression of apoptosis related genes was determined by qRT-PCR. The qRT-PCR was performed using the Prism 7500 Sequence Detection System; (**d**) The activity of caspase-9 and caspase-3 was measured by using a protein activity assay; and, (**f**) Western blotting technique detected the effect of 20(S)-GRh2 on the apoptosis-related proteins (caspase-3, PARP). The loading control protein is β-actin. Values shown in (**a**–**f**) are mean ± SD; (**a**–**f**) are the representative experiment with *n* = 3 technical replicates from two independent experiments with similar results. * *p* < 0.05 and vs. U937 cell control group; # *p* < 0.05 and vs. K562 cell control group.

The ROS act as an apoptotic signal mediator in the apoptosis [37]. ROS play a critical role in mediating the cytotoxicity that is induced by many natural chemotherapeutic agents, including 20(S)-GRh2. Therefore, the effects of 20(S)-GRh2 on ROS generation by using Cellular ROS Detection Assay Kit in K562 and U937 cells were then examined. 20(S)-GRh2 treatment induced ROS generation in a dose-dependent manner (Figure 2a). The production of ROS was 1.8 (3.1) in U937 cells and 2.4 (4.6) fold in K562 cells than the control group, which indicated that K562 could generate more ROS, when treated with the concentrations of 60 (80) μM 20(S)-GRh2. It is known that the MMP disruption could be observed during the effector phase of apoptosis, which can induce the release of pro-apoptosis related factor and active caspase related protein, which leads to the final death [38]. We further analyzed ∆ψm by JC-1 molecular probes, where the decreasing relative proportion of red and green fluorescence were used to measure the mitochondrial depolarization. The observed results have been shown in Figure 2b, where the level of MMP was 0.62 (0.34) in U937 cells and 0.43 (0.24) folds in K562 cells than control group, which manifested that the decline of MMP was greater in K562, when incubated with the concentrations of 60 (80) μM in U937 and K562 cells. The above results indicated a higher ROS content and a lower level of MMP in K562 than U937 cells under the treatment of 20(S)G-Rh2.

Generally, in most tumor cells, the mitochondrial membrane potential is controlled by anti-apoptotic genes (e.g., bcl-2, bcl-X_L_) and pro-apoptotic genes (i.e., bax) [39]. The mitochondrial apoptosis related gene and protein have been discussed. The expression of bcl-2 family related genes and caspase genes are the key to regulating apoptosis, including anti-apoptotic genes (bcl-2, bcl-X_L_) and pro-apoptotic genes (bax). In order to explore the molecular mechanism of 20(S)-GRh2, the expression of bcl-2, bcl-X_L_, and bax was measured and the obtained results have been shown in Figure 2c, where the ratio of bax/bcl-2(bcl-xl) were 1.9 (2.4) in U937 cells and 6.1(4.6) in K562 cells that were treated by 80 μΜ 20(S)-GRh2, which indicated that the ratio of bax/bcl-2(bcl-xl) increased more in K562 cells when being treated with the same concentration.

The mitochondrial apoptosis related caspase proteins (caspase-3, caspase-9) were enhanced in both gene expression and protein activity with increasing concentrations of 20(S)-GRh2 in U937 and K562 cells (Figure 2c–e), where the activation of caspase related proteins (caspase-3, caspase-9) were higher in K562 than U937 cells. The immunofluorescence analysis also revealed the cleavage of caspase-3 was 1.8 and 3.6 folds in U937 cells and 4.7 and 6.2 folds in K562 cells than control group; Also, the cleavage of PARP was 1.3 and 2.4 fold in U937 cells and 2.0 and 4.1 fold in K562 cells than the control group, which indicated that K562 could induce more cleavage of caspase-3 and PARP when treated with the concentration of 60 (80) μM of 20(S)-GRh2 (Figure 2f). These outcomes are in consistent with the greater apoptosis, resulting in K562 than U937 cells.

### 3.3. 20(S)-GRh2 Induces Autophagy in Myeloid Leukemia Cells 

**Figure 3:**
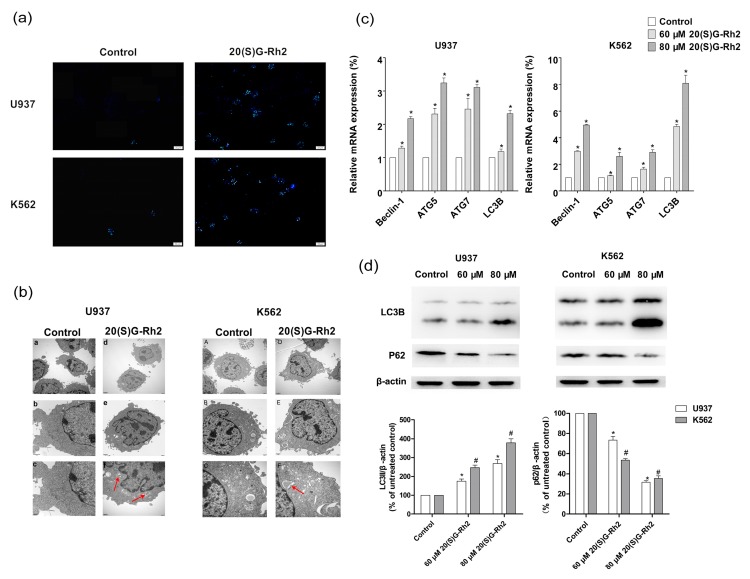
20(S)-GRh2 induced autophagy in U937 and K562 cells. (**a**) MDC staining represents autophagic vacuole by fluorescence microscope (Bar = 20 μM); (**b**) TEM observations demonstrate 20(S)-GRh2-induced autophagy. The arrows indicate intracellular double-membrane vesicles. Bar, 1 μm in (A and a); 2 μm in (B and b); 500 nm in (C and c); (**c**) The expression of autophagy related genes was determined by qRT-PCR. The qRT-PCR was performed using the Prism 7500 Sequence Detection System; (**d**) The expression of autophagy related proteins, LC3B and p62 were determined by a western blot in the presence of 20(S)-GRh2. β-actin was used as an internal control. The loading control protein is β-actin. Values shown in (**c**) are mean ± SD; (**a**–**d**) are the representative experiment with *n* = 3 technical replicates from two independent experiments with similar results. * *p* < 0.05 and vs. U937 cell control group; # *p* < 0.05 and vs. K562 cell control group.

To examine whether 20(S)-GRh2 induces autophagy in U937 and K562 cells, MDC staining and TEM assays were used to detect the ultrastructural features of autophagy in these cells. The obtained results showed that 20(S)-GRh2 induced the generation of acidic vesicular organelles (AVOs) (Figure 3a). Furthermore, TEM results also demonstrated an autophagic vacuoles that were surrounded by double-membrane structures significantly increased in K562 and U937 cells when treated with 20(S)-GRh2 (Figure 3b). The autophagy related genes (Beclin-1, ATG5, ATG7, LC3B) were increased both in U937 and K562 cells (Figure 3c). Autophagosome formation involves the conversion of LC3 from the cytosolic LC3-I to the autophagosome-associated LC3-II form and the downstream degradation of autophagy related protein will lead to the accumulation of p62, which indicates the autophagic flux [40]. In Figure 3d, the conversion of LC3I to LC3II was 1.7 (2.6) fold in U937 cells and 2.4 (3.7) fold in K562 cells than the control group, where the expression of p62 was 0.7 (0.3) fold 0.5 (0.3) than the control when treated with the concentration of 60 (80) μM of 20(S)-GRh2. The observed results demonstrate that the treatment of U937 and K562 cells with 20(S)-GRh2 induced the conversion from LC3-I to LC3-II, and decreased the expression of p62 protein in a dose-dependent manner.

### 3.4. Effect of Autophagy Inhibitor (3-MA) on 20(S)-GRh2 Induced Apoptosis and Autophagy

**Figure 4:**
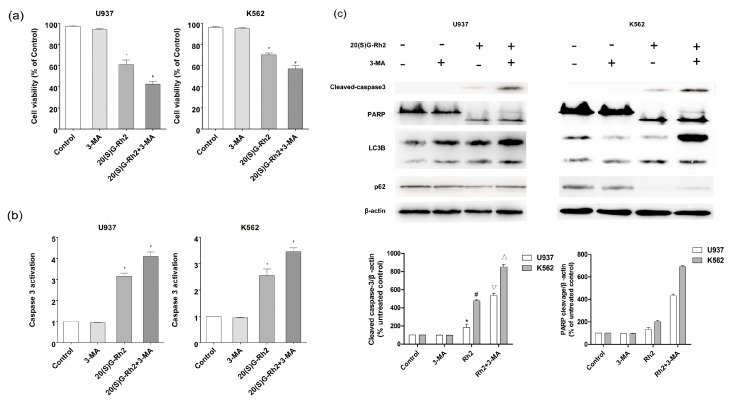
Effect of autophagy inhibitor 3-MA on 20(S)-GRh2 induced apoptosis and autophagy in U937 and K562 cells. (**a**) Cell viability was measured by CCK8 assay; (**b**) The activation of caspase-3 was measured by caspase-3 Fluorescent activity Kit; (**c**) The level of apoptosis related proteins (caspase-3 and PARP) and autophagy related proteins (LC3B, P62) were detected by western blot under the treatment of 20(S)-GRh2. β-actin was used as an internal control. The loading control protein is β-actin. The experimental cells were treated with 20(S)-GRh2, Z-VAD-FMK, 20(S)-GRh2 + Z-VAD-FMK (the cells were pretreated with Z-VAD-FMK followed by exposing to 20(S)-GRh2), control cells were cultured in 0.5% DMSO medium only. Values shown in (**a**–**c**) are mean ± SD. (**a**–**c**) are the representative experiment with *n* = 3 technical replicates from two independent experiments with similar results. * *p* < 0.05 and vs. U937 cell control group; # *p* < 0.05 and vs. K562 cell control group; ∇ *p* < 0.05 vs. 20(S)-GRh2 group in U937 cells; △ *p* < 0.05 vs. 20(S)-GRh2 group in K562 cells.

In order to explore the relationship between autophagy and apoptosis, an autophagy inhibitor was used to pretreat the cells before subjecting to 20(S)-GRh2 treatment. As shown in Figure 4a, the cell viability decreased both in U937 and K562 cells when pretreated with 3-MA. Furthermore, the activation of caspase-3 protein was improved) when it was pretreated with 3-MA (Figure 4b). In Figure 4c, the cleavage of caspase-3 in the 20(S)-GRh2 + 3MA group (5.3 fold and 8.5 fold) was higher than the 20(S)-GRh2 (1.8 fold and 4.7 fold) in U937 and K562 cells respectively, the cleavage of PARP in the 20(S)-GRh2 + 3MA group (4.3 fold and 6.9 fold) was higher than the 20(S)-GRh2 (1.3 fold and 4.0 fold) in U937 and K562 cells, respectively. The western blotting analysis demonstrated that 3-MA increased the cleavage of caspase-3 and PARP. Meanwhile, the conversion of LC3I to LC3II was decreased and the expression of p62 in U937 cells and K562 cells was enhanced. These data indicate that the 20(S)-GRh2-induced autophagy plays a role in cell protection against apoptotic cell death. 

### 3.5. Effect of Apoptosis Inhibitor (Z-VAD-FMK) on 20(S)-GRh2 Induced Autophagy and Apoptosis

**Figure 5:**
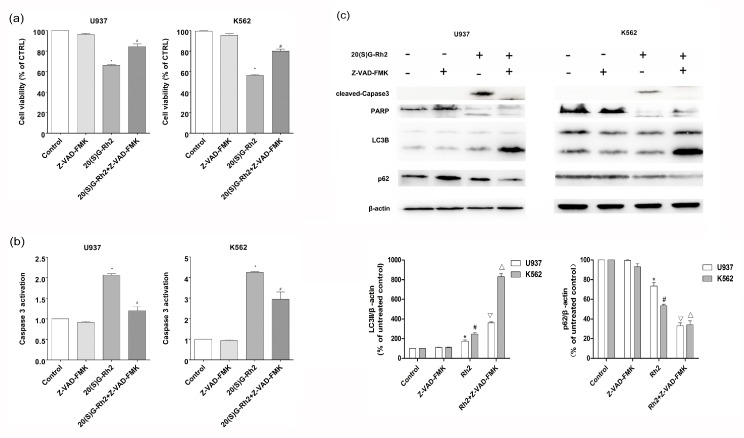
Effect of apoptosis inhibitor on 20(S)-GRh2 induced apoptosis and autophagy in U937 and K562 cells. (**a**) Cell viability was measured by CCK8 assay; (**b**) The activation of caspase-3 was measured by caspase-3 Fluorescent activity Kit; (**c**) The level of autophagy related proteins (LC3B, P62) and apoptosis related proteins (caspase-3 and PARP) was detected by western blot under the treatment of 20(S)-GRh2. β-actin was used as an internal control. The experimental cells were treated with 20(S)-GRh2, Z-VAD-FMK, 20(S)-GRh2 + Z-VAD-FMK (the cells were pretreated with Z-VAD-FMK followed by exposing to 20(S)-GRh2), control cells were cultured in 0.5% DMSO medium only. Values shown in (**a**–**c**) are mean ± SD. (**a**–**c**) are the representative experiment with *n* = 3 technical replicates from two independent experiments with similar results. * *p* < 0.05 and vs. U937 cell control group; # *p* < 0.05 and vs. K562 cell control group; ∇ *p* < 0.05 vs. 20(S)-GRh2 group in U937 cells; △ *p* < 0.05 vs. 20(S)-GRh2 group in K562 cells.

The Z-VAD-FMK pan-caspase inhibitor was used as an apoptosis suppressor to further determine the relationship between apoptosis and autophagy induced by 20(S)-GRh2. As shown in Figure 5a, Z-VAD-FMK strongly restored the cell viability after 20(S)-GRh2 treatment for 24 h in U937 and K562 cells. Additionally, Z-VAD-FMK markedly decreased the activation of caspase-3 (Figure 5b). In Figure 5c, the conversion of LC3I to LC3II in the 20(S)-GRh2 + Z-VAD-FMK group (3.6 fold and 8.3 fold) was higher than the 20(S)-GRh2 (1.7 fold and 2.5 fold) in U937 and K562 cells, respectively, where the expression of p62 in the 20(S)-GRh2 + Z-VAD-FMK group (0.33 fold and 0.39 fold) was lower than the 20(S)-GRh2 (0.7 fold and 0.5 fold) in U937 and K562 cells, respectively. The pretreatment of Z-VAD-FMK increased the conversion of LC3I to LC3II and inhibited the expression of p62, indicating that the inhibition of apoptosis diminished the autophagy that was induced by 20(S)-GRh2.

## 4. Discussion

As a traditional Chinese medicine, Panax ginseng has been used in eastern Asia for thousands of years. 20(S)-GRh2 is the main bioactive component in ginseng extracts, which exhibits a potent ability in killing the cancer cells [17]. Some studies have reported that treating with 20(S)-GRh2 could be a promising therapy for cancers [41]. In recent years, the drugs which target autophagy pathway demonstrate promoting the autophagic death of tumor cells or inhibiting the autophagic protection in order to eliminate tumor cells and resistance to chemotherapy, which overcome the tumor problems and bringing a glimmer of hope in cancer therapy [42]. On the other hand, many studies have reported that 20(S)-GRh2 has a significant apoptosis effect for K562 and U937cells [19,20,21,43,44,45]. Nevertheless, the autophagy that is induced by 20(S)-GRh2 in K562 and U937 cells, and the difference between the two cells to the 20(S)-GRh2 induced apoptosis was not reported. Meanwhile, the relationship on the apoptosis and autophagy of 20(S)-GRh2 in these two cells were not studied. In the current research, we firstly reported that 20(S)-GRh2 could induce the apoptosis in the two cells and the phenomenon was more obvious in K562 than U937 cells. Moreover, the 20(S)-GRh2 could trigger the autophagy in K562 and U937 cells. Finally, it has been found that apoptosis and autophagy could act as partners to induce cell death in a cooperative manner.

CCK-8 assay (Figure 1b) has demonstrated that U937 and K562 cells both exhibited—proliferation inhibition to 20(S)-GRh2 with an IC50 of 60 μM and 80 μM. K562 exhibited an inhibition of the proliferation to 20(S)-GRh2 with an IC50 of 60 μM, which is a little potent than that reported by Liu et al. (80 μM) [21]. U937 exhibited inhibition of the proliferation to 20(S)-GRh2 with an IC50 of 80 μM, which is more potent than reported by Wang et al. (160 μM) [46]. For U937 cells, the IC50 reported in the literature was in the 16–160 μM [46] and the IC50 for K562 cells is in the 20–80 μM [21]. This shows that the dose we used is reasonable. Previous studies have also demonstrated that 20(S)-GRh2 (60/80 μM) exhibited low toxicity on non-cancerous cells (normal bone marrow) [21,22]. To further explore the mechanism of different proliferation inhibition in the two cells, the morphology and flow cytometry were used. Cell apoptosis has a number of biochemical features, including cell crinkles, membrane bubbles, chromosomal condensation (nuclear sequestration), nuclear fragmentation, and structural disassembly [42]. There are studies that revealed that 20(S)-GRh2 could induce apoptosis in Kasumi-1, HL60 cells [22]. Our Hoechst 33342 staining results also found typical morphological features of apoptotic cells and apoptosis assay results indicated a higher percentage of apoptotic cells in K562 cells than in U937 cells when these cells were treated with 20(S)-GRh2 (Figure 1a,c). 20(S)-GRh2 induced apoptosis through mitochondrial signaling pathways [18]. 20(S)-GRh2 induced ROS, which as a positive feedback mechanism to activate ROS release from neighboring mitochondria, is an important signaling intermediates leading to depolarization of the mitochondrial membrane potential that release the pro-apoptosis factor (cytochrome, caspase-9) and result in 20(S)-GRh2 induced apoptosis [47,48,49]. In this study, we also tested the role of ROS in the two cells. Our date showed that 20(S)-GRh2 could also trigger the production of ROS and the higher content of ROS was achieved in K562 cells than in U937 cells. On the other hand, excessive ROS might cause the increased permeation of mitochondrial membrane and result in the disappearance of MMPs, which is widely considered as one of the earliest events in the process of apoptosis [16,49,50]. In Figure 2a,b, we showed that the level of MMP was lower in K562 cells than in U937 cells. It is known that the higher ROS causes an increased reduction of MMP. The results of MMP were consistent with the different content of ROS. Generally, in most of the tumor cells, the mitochondrial membrane potential is controlled by anti-apoptotic genes (e.g., bcl-2, bcl-xl) and pro-apoptotic genes (i.e., bax). The increased ratio of bax/bcl-2(bcl-xl) controls the mitochondrial membrane permeability [45,51,52]. In Figure 2c, the higher ratios were achieved in K562 cells than in U937 cells, which was consistent with a change in MMP. The change of MMP further triggers the release of apoptosis factor, and activates the caspase related proteins, caspase-9 and caspase-3 [18]. Activated caspase-3 could further cleave specific substrates, such as PARP protein, which could lead to cell death finally. Meanwhile, we indeed found out a higher activation of caspase-3 and caspase-9, as shown in Figure 2d,e as well as the cleavage of Caspase-3 and PARP as indicated in Figure 2f in K562 cells than in U937 cells. All these results demonstrated that the K562 cells and U937 cells exhibit significant proliferation inhibition with the treatment of 20(S)-GRh2 through the level of ROS, MMP, and expression of gene and protein.

Autophagy is a natural, destructive cellular mechanism that could degrade damaged proteins and cytoplasmic components of lysosomes autonomously, thus maintaining cellular homeostasis and supplying pre-substrate energy in cells [29]. MDC staining and TEM both displayed the ultrastructural features of autophagy in these cells (Figure 3a,b). The results of genes showed that the expression of Atg 5, Atg 7, and beclin-1 was increased after treating with 20(S)-GRh2 in U937 and K562 cells (Figure 3c), and were consistent with the previous studies [53,54,55]. LC3, which is a recognized indicator of autophagy evaluation and measurement, is widely used by many autophagy researchers. During autophagy, the microtubule-associated protein light chain 3 (MAP-LC3) in the cytoplasm would cleave off a small segment of the polypeptide to form LC3-I. LC3-I conjugates with phosphatidylethanolamine (PE) to form an autophagosome membrane (LC3-II). Therefore, the magnitude of LC3-II/I ratio can be used to estimate the level of autophagy [56]. The enhancement or downstream degradation of autophagy will lead to the accumulation of p62. p62 will eventually mature into autophagy in vivo, and degradation in autophagy, and thus p62 levels are negatively related to autophagy [11,12]. Our results showed a conversion from LC3-I to LC3-II and a decrease in the expression of p62 after the cells were treated by 20(S)-GRh2 (Figure 3d), which indicate that the autophagy phenomenon is increased with an increase in the dose of 20(S)-GRh2 in U937 and K562 cells.

Autophagy and apoptosis are important to control the cell death and there is a complex relationship between autophagy and apoptosis. Meanwhile, the relationship is different and which depends upon the cell lines [29]. Autophagy blocks the induction of apoptosis in general and apoptosis-associated caspase activation turns off the autophagic process. However, in some circumstances, autophagy may help to induce apoptosis and lead to autophagic cell death [57]. Previous studies have reported that 20(S)-GRh2 displays its inhibitory effect on the cells through a combination of apoptosis and autophagy; nevertheless, there is no investigations that have reported between their relationship in K562 and U937 cells [3,19].

Research has shown that autophagy, as a survival mechanism of cancer cells, not only provides energy during stress, but also protects cancer cells that are treated with anti-cancer measures from apoptosis [29]. In our study, we treated the cells with autophagy inhibitor, 3-MA, and apoptosis inhibitor, Z-VAD-FMK to observe the phenomenon of apoptosis and autophagy. When we pretreated the cells with 3-MA, we found that 3-MA enhanced 20(S)-GRh2 induced caspase-3 and PARP protein cleavage (Figure 4c). This demonstrated that autophagy inhibition enhanced 20(S)-GRh2-induced cell apoptosis in U937 and K562 cells. Meanwhile, when we pretreated with an apoptosis inhibitor, Z-VAD-FMK, also decreased the expression level of P62, and increased the conversion of LC3I to LC3-II (Figure 5c). These results indicate that 20(S)-GRh2 in U937 and K562 cells could induce autophagy and apoptosis, and the two processes are in a cooperative manner.

## 5. Conclusions

In summary, for the first time, the results that were obtained through the present investigation revealed that 20(S)-GRh2 displays its anti-cancer effects, which are regulated by apoptosis and autophagy. Meanwhile, we further demonstrated that 20(S)-GRh2-induced relationship between apoptosis and autophagy is cooperative in K562 and U937 cells. This research may provide a rationale for future clinical application using 20(S)-GRh2 as a chemotherapeutic agent for AML and CML. We could also assume that the inhibition of autophagy could be a potential therapeutic strategy to improve the efficiency of 20(S)-GRh2.
